# cGAS-dependent proinflammatory and immune homeostatic effects of the microtubule-targeting agent paclitaxel

**DOI:** 10.3389/fimmu.2023.1127623

**Published:** 2023-03-07

**Authors:** Angela Flavia Serpico, Caterina Pisauro, Domenico Grieco

**Affiliations:** ^1^ CEINGE Biotecnologie Avanzate Franco Salvatore, Naples, Italy; ^2^ Dipartimento di Medicina Molecolare e Biotecnologie Mediche (DMMBM), University of Naples “Federico II”, Naples, Italy

**Keywords:** microtubule-targeting agents, Paclitaxel, cGAS, STING, IFN, PD-L1, immune checkpoint inhibitors

## Abstract

Taxanes are Microtubule-Targeting Agents (MTAs) that exert potent anticancer activity by directly killing cancer cells. However, recent evidence suggests that they may also stimulate inflammation and anticancer adaptive immunity and that these actions strongly contribute to their therapeutic efficacy. Details on how Taxanes may modulate inflammation and anticancer immunity are, nevertheless, still missing. We show here that at very low doses the Taxane Paclitaxel (Pxl) indeed induces a potent proinflammatory response in various cancer cell types in a cyclic GMP-AMP (cGAMP) synthase (cGAS)- and Stimulator of Interferon Genes (STING)-dependent manner, leading to interferon (IFN) signaling. However, we find that Pxl treatment also strongly upregulates the expression of the immune checkpoint protein Programmed Death-Ligand 1 (PD-L1) in cancer cells, therefore, inducing an inhibitory response to adaptive immunity potentially attenuating anticancer immunity and therapeutic success. These observations provide a mechanistic explanation of why clinical benefit may derive from the combination of Pxl with Immune Checkpoint Inhibitors (ICIs) and suggest that more accurately tailoring dosage and schedule of this combination therapy may provide benefit in the management of a larger number of cancer types and stages.

## Introduction

Microtubule-Targeting Agents (MTAs) are very widely used and effective anticancer drugs. In particular, the microtubule stabilizer MTA Paclitaxel (Pxl) is used for the therapy of a variety of different cancers. By perturbing microtubule dynamics, Pxl affects mitotic progression by activating the spindle assembly checkpoint (SAC), a safeguard mechanism that delays mitosis exit when spindle assembly is incomplete or abnormal (1–3). Prolongation of mitosis may result in activation of apoptotic pathways and efficient cancer cell killing, however, cancer cells can adapt to the SAC and slip through an abnormal mitosis and this may result in cancer resistance to Pxl treatment (4). Cancer cell response to Pxl appears, therefore, somewhat variable and evidence also suggests that the therapeutic efficacy of Pxl correlates with induction of chromosome segregation defects rather than with delayed mitosis (5).

Chromosome segregation errors are often accompanied by the formation of micronuclei (1). Indeed, chromosomes that are segregated asynchronously relatively to the majority will be enveloped in a nuclear membrane separated from that of the main nucleus, forming micronuclei (1). In addition, micronuclear membrane can be different from the membrane of the main nucleus and micronuclear DNA can undergo damage, fragmentation and rearrangements, a phenomenon called chromothripsis (6). While chromothripsis may be a mechanism that increases genome instability and fuels carcinogenesis, endogenous damaged DNA, altered micronuclear membrane as well as DNA bridges resulting from abnormal chromosome segregation induced by Taxanes also promote activation of the cytosolic DNA sensor cyclic GMP-AMP (cGAMP) synthase (cGAS) (1, 7, 8). The cGAS enzyme was originally identified as an important regulator of the innate immunity in response to infection because of its ability to recognize pathogen DNA in the cytoplasm of an infected cell (9). More recently, cGAS has been shown to be activated also by binding to endogenous cytosolic DNA fragments, as in senescent or damaged cells, or to endogenous DNA within micronuclei that form in cells following chromosome segregation errors (8–11). Indeed, the membrane of micronuclei is often prone to breakage, due to alterations in assembly of the micronuclear lamina, and broken micronuclear membrane causes cytosolic exposure of micronuclear DNA that can consequently interact with cGAS and activate it (7–11).

Active cGAS generates the cyclic dinucleotide cGAMP that, acting as a second messenger, binds the adaptor protein Stimulator of Interferon Genes (STING), resident in the endoplasmic reticulum (9, 11, 12). cGAMP binding to STING promotes conformational changes in STING that allows its interaction with TANK-binding kinase 1 (TBK1), resulting in TBK1 activation (9). Active TBK1 phosphorylates and activates the Interferon Regulatory Factor 3 (IRF3) leading to upregulation of expression of interferons (IFNs) and promoting the inflammatory response (9, 11–13). The inflammatory response and IFN-pathway activation may further call in the intervention of the adaptive immune system (13). These observations have led to the hypothesis that Taxane-based cancer therapy may also work because it promotes inflammation, rendering “hot” the tumor microenvironment, and favoring the intervention of the adaptive immune system to kill cancer cells (1, 14, 15).

Here, by analyzing the effect of low doses of Pxl in various cancer cell lines, we show that Pxl treatment indeed results in a cGAS-dependent activation of a proinflammatory cascade. However, we also find that Pxl stimulates, in a cGAS-dependent fashion, upregulation of the expression of the immune checkpoint protein PD-L1 in cancer cells that, on the other hand, may favor cancer cell evasion form immunosurveillance. On the basis of these findings, we propose that Plx treatment may prime cancer cells to susceptibility to therapy with Immune Checkpoint Inhibitors (ICIs) and that the timing and dosage of the combination therapy of Pxl, and possibly other Taxanes, with ICIs may strongly affect treatment efficacy.

## Materials and methods

### Cell lines and cell culture

A549, HeLa, MCF7, MDA-MB231cells were form CEINGE Cell Culture Facility. A549 cells were grown in Roswell Park Memorial Institute Medium - 2% L-glutamine (RPMI-1640; Cat# R8758; Sigma-Aldrich, St. Louis, MO, USA), HeLa cells were grown in Dulbecco’s Modified Eagle Medium - high glucose (DMEM; Cat# D6429; Sigma-Aldrich), MCF7 cells were grown in Minimum Essential Medium - 2% L-glutamine (MEM; Cat# M4655; Sigma-Aldrich), MDA-MB231 cells were grown in Dulbecco’s Modified Eagle Medium – low glucose (DMEM; Cat# D6046; Sigma-Aldrich), all supplemented with 10% fetal bovine serum (FBS; Cat# CHA30160L; GE Healthcare Life Sciences, Pittsburgh, PA, USA), 1% penicillin/streptomycin (Cat# ECB3001D; Euroclone, Pero, MI, Italy), and incubated in a humidified incubator at 37° C with 5% CO_2_.

### Cell treatments and chemicals

For biochemical and immunofluorescence studies, cells were seeded at a cell density of 7000/cm^2^ either into 10 cm dishes or onto glass coverslips and treated with vehicle, dimethylsulfoxide (DMSO), as control or Paclitaxel (Plx; Cat# T1912; Calbiochem, Merck Millipore, Billerica, MA, USA) at the indicated doses, after 24 hours from seeding. Cell samples were taken at the indicated time points, washed once in PBS (Cat# ECB4004LX; 10Euroclone) and lysed with 5 volumes of lysis buffer (LB; 0.2% Igepal; 80 mM b-glycerophosphate, 10 mM MgCl_2_, 20 mM EGTA, 250 mM NaCl; Sigma-Aldrich) or fixed as described in Immunofluorescence fixation and staining section. Lysates were incubated 30 min on ice and then spun for 20 min at 13.200 rpm in a refrigerated microcentrifuge (4°C; Eppendorf centrifuge 5424R). For cell counting and viability assays, cells were seeded at 7000 cells/cm2 density. After 24 hours of incubation, one cell sample per cell type was trypsinized, collected and resuspended in PBS and loaded into a Bürker counting chamber, cells were counted manually under a microscope for the Time 0 cell count. Other cell samples were treated with either vehicle (DMSO) as control or with 2, 4 and 8 nM Plx and incubated for 48 hours, then cells were trypsinized and resuspended in PBS, mixed to an equal volume of Trypan Blue and counted (Trypan Blue solution 0.4%; Cat# 15250061; Gibco - Thermo Fisher Scientific, Waltham, MA, USA). Cell counting was conducted manually under a microscope and cell viability was determined by Trypan Blue exclusion.

### RNA interference

RNA interference *via* siRNAs was performed using DharmaFECT 1 siRNA Transfection Reagent (Cat# T200103; Dharmacon). For efficient knock-down cells were plated 24 hours prior to treatment and transfected with 25 nM of non-targeting or targeting siRNAs duplex using DharmaFECT 1, according to the manufacturer’s protocol. DharmaFECT 1 and siRNAs were mixed in RPMI-1640 medium and incubated at room temperature (rt) for 20 min, then, the mixture was added to the cells and incubated for 24 hours before Paclitaxel addition. Non-targeting or human cGAS-targeting siRNAs (Non-Targeting SMARTpool Cat# L-009326-00-0020; MB21D1-Targeting SMARTpool Cat# L-015607-02-0020) were purchased from Dharmacon.

### Immunoblotting

Immunoblotting was performed as described (16). Briefly, samples were prepared by adding SDS loading buffer (Laemmli sample buffer; Cat# 1610747; BioRad, MI, Italy) to lysates. Samples were boiled for 10 min at 99°C before being separated on SDS-PAGE (poly-acrylamide percentage spanning from 10 to 12%). Proteins were blotted onto nitrocellulose membrane (Cat# GEH10600002; GE Healthcare) using a wet-transfer system (Cat# EI0001; ThermoFisher). Membranes were incubated with 5% not fat dry milk (NFDM; Cat# A0830; AppliChem GmbH, DA, Germany) or 3% bovine serum albumin (BSA; Cat# A7030; Sigma-Aldrich) in PBS or TBS (tris buffered saline; Cat# T5912; Sigma-Aldrich) supplemented with 0.01% Tween20 (Cat# P9416; Sigma-Aldrich; TPBS or TTBS, respectively) for 1 hour at rt. Then, membranes were incubated with primary antibodies, diluted in TPBS or TTBS, at 4°C overnight. After washing twice with TPBS or TTBS, filters were incubated with secondary peroxidase-conjugated antibodies, diluted in TPBS or TTBS, for 1 hour at rt. Detection was performed using an Enhanced ChemiLuminescence (ECL) kit (Cat# GEHRPN2106; GE Healthcare). Blots were acquired using Canon CanoScan LiDE 300 scanner (Canon) and scanned at 300 dpi. Primary and secondary antibodies used for immunoblotting are listed in **Table 1**.

**Table 1:** Antibodies used in this study.

Antibodies	Source	Catalog number
Primary		
rabbit anti-cGAS	Cell Signaling Technology, Danvers, MA, USA	15102S
mouse anti-α-tubulin	Sigma-Aldrich, St. Louis, MO, USA	T9026
rabbit anti-phospho-serine-366-STING (p-S366-STING)	Cell Signaling Technology	50907S
rabbit anti-STING	Cell Signaling Technology	13647S
rabbit anti-phospho-serine-386-IRF3 (p-S386-IRF3)	Thermo Fisher Scientific, Waltham, MA, USA	PA5-99387
rabbit anti-IRF3	Cell Signaling Technology	4302S
rabbit anti-phospho-tyrosine-701-STAT1 (p-Y701-STAT1)	Cell Signaling Technology	9167S
rabbit anti-STAT1	Cell Signaling Technology	14994S
mouse anti-γ-tubulin	Sigma-Aldrich	T5326; Clone GTU-88
rabbit anti-PD-L1	Cell Signaling Technology	15165S 86744S
Secondary		
sheep anti-mouse IgG HRP linked	GE Healthcare Life Sciences, Pittsburgh, PA, USA	NA931
donkey anti-rabbit IgG HRP linked	GE Healthcare	NA934
goat anti-mouse IgG Alexa Fluor 488	Thermo Fisher Scientific	A11029
donkey anti-rabbit IgG Alexa Fluor 594	Thermo Fisher Scientific	A21207

### Immunofluorescence

Cells were plated onto glass coverslips 24 hours prior to Paclitaxel addition. After 48 hours Plx treatment, coverslips were briefly washed in PBS and cells fixed with 4% paraformaldehyde (Cat# P6148; Sigma-Aldrich) in PBS (Euroclone) for 10 min at rt. Cells were washed twice with PBS and permeabilized with 0.25% Triton X- 100 (Cat# T9284; Sigma-Aldrich) in PBS for 15 min. The permeabilization step was omitted for PD-L1 immunofluorescence. Then, cells were washed once with PBS and incubated with 3% bovine serum albumin (BSA; Sigma-Aldrich) in PBS for 1 hour at rt. Coverslips were transferred into a humidity chamber and incubated with primary antibodies in 1.5% (w/v) BSA-PBS solution for 2 hours at rt, except for PD- L1 immunofluorescence for which incubation with primary antibody was performed overnight at 4°C. After incubation, cells were washed three times with PBS and incubated with fluorescently labelled secondary antibodies, diluted in 1.5% BSA-PBS solution, for 1 hour at rt. DNA was stained with Hoechst 33258 (1 mg/mL; Cat# 94403; Invitrogen, Waltham, MA, USA) by incubation for 10 min. Finally, cells were washed four times with PBS and slides mounted with Mowiol 40-88 (Cat# 81381; Sigma-Aldrich). Primary and secondary antibodies used for immunofluorescence are listed in **Table 1**.

### Microscopy

Fixed cells were photographed using an inverted confocal fluorescence microscope LSM 980 (Zeiss) equipped with a 63X/1.4 oil objective (Zeiss). Representative images were obtained collecting 5 Z-stack series. The acquisitions were deconvoluted and projected into one plane using the ZEN3.1 software.

## Results

### Pxl treatment induces formation of cGAS-positive micronuclei

It has been proposed that chromosome segregation defects in chromosomally unstable cancer cells or upon treatment of cancer cells with Taxanes can cause formation of cGAS-positive micronuclei and activation of proinflammatory pathways (1, 15, 17). To investigate potential immunomodulatory effects of the Taxane Pxl, we started asking whether treatment with low doses of Pxl promoted formation of cGAS-positive micronuclei in various cancer cell lines: lung adenocarcinoma A549 cells, triple negative breast cancer (TNBC) MDA-MB231 cells, hormone-responsive breast cancer MCF7 cells and cervical cancer HeLa cells. We chose a low Pxl concentration range, between 2 and 8 nM, because it is known that Pxl concentrates within cells about 50 to 1000 folds, depending on the cell type, so that after a 20-hour treatment with Pxl concentrations in the low nM range in the cell culture medium, Pxl can reach intracellular concentrations similar to those reached, *in vivo*, in tumor cells of patients treated with Pxl infusions (5). In addition, since Pxl-induced micronucleation requires passage through mitosis, we chose a treatment time of 48 hours since, under our experimental conditions, the cell doubling time was approximately 22 hours for A549, 24 hours for HeLa, 27 hours for MCF7 and 29 hours for MDA-MB231 in control cells (treated just with dimethylsulfoxide, DMSO, vehicle; **Supplementary Figure 1**), so that by 48 hours all cell types had undergone mitosis at least once (1, 5). Moreover, a 48-hour Pxl treatment had mostly a cytostatic effect at the doses used in all cell lines tested, while cytotoxicity was relatively modest since the highest drop of cell viability (measured by trypan blue exclusion) was around 20% in MDA-MB231 cells treated with 8 nM Pxl (**Supplementary Figure 1**).

**Figure 1:**
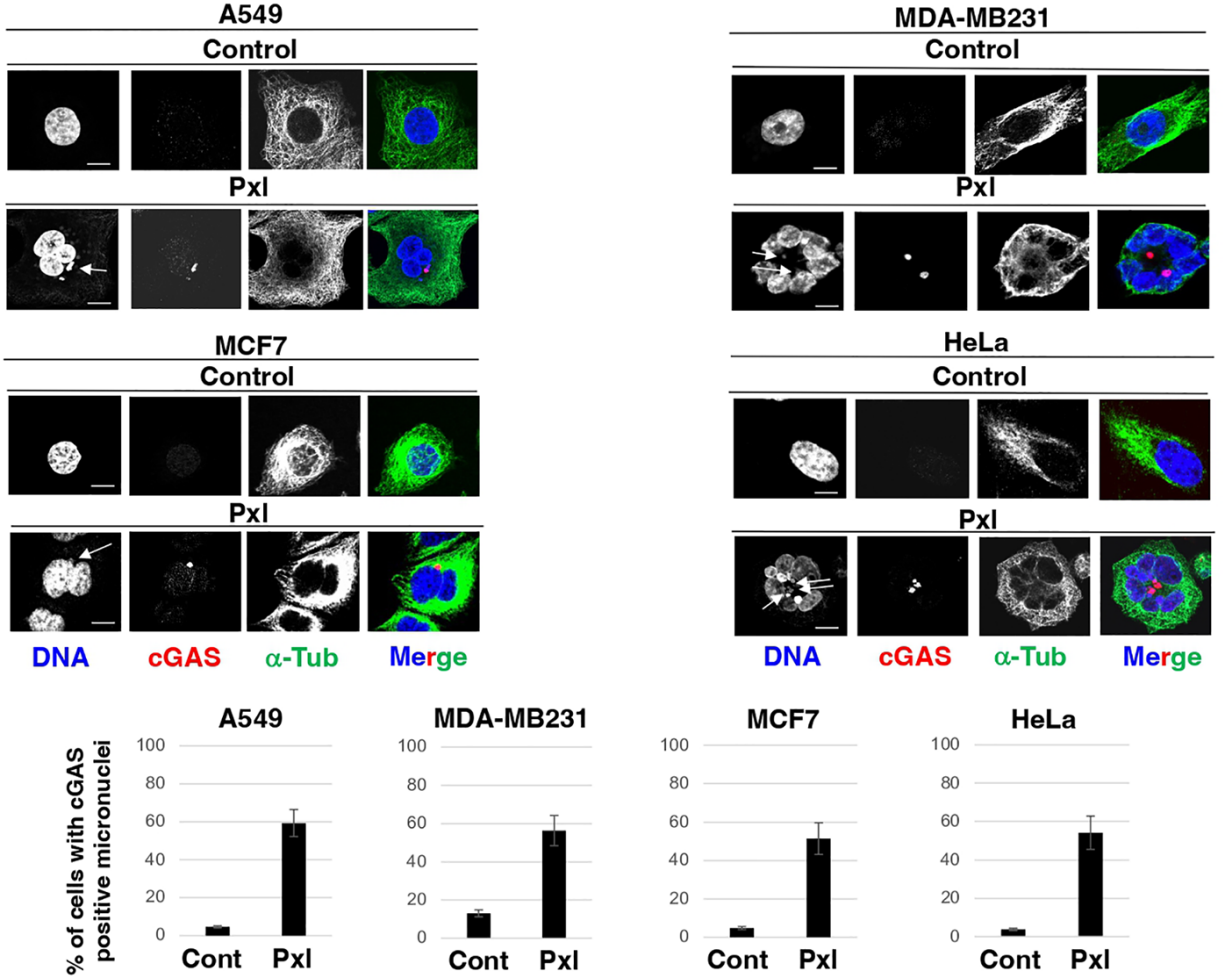
Pxl increases cGAS-positive micronuclei formation. A549, MDA-MB231, MCF7 and HeLa cells were treated for 48 hours with either vehicle (DMSO) as control or Pxl (4 nM), fixed and processed for indirect immunofluorescence (IF) staining for the indicated antigens. Representative IF images are shown. Scale bar: 5 μm. Lower graphs show a quantitation of cGAS-positive micronuclei (error bars refer to variability within three independent experiments; around 200 cells were scored for control and Pxl treatment per experiment).

Thus, to determine whether Pxl treatment promoted formation of cGAS-positive micronuclei, the four cell lines were treated with vehicle, as control, or with 4 nM Pxl for 48 hours and the presence of cGAS-positive micronuclei assessed by immunofluorescence (IF; **Figure 1**). In all cell lines tested, including MDA-MB231 that are very genetically unstable and show a higher basal level of cGAS-positive micronuclei, Pxl treatment strongly increased the number of cells with cGAS-positive micronuclei (**Figure 1**).

Chromatin bridges that form during altered mitosis exit induced by Pxl have also been shown to recruit cGAS and activate the cGAS-STING pathway involved in activation of cGAS (17). We also found evidence of Pxl-induced cGAS-positive chromatin bridges under our experimental conditions (an example is shown in **Supplementary Figure 2**).

### Pxl induces a proinflammatory cascade in cancer cells

**Figure 2:**
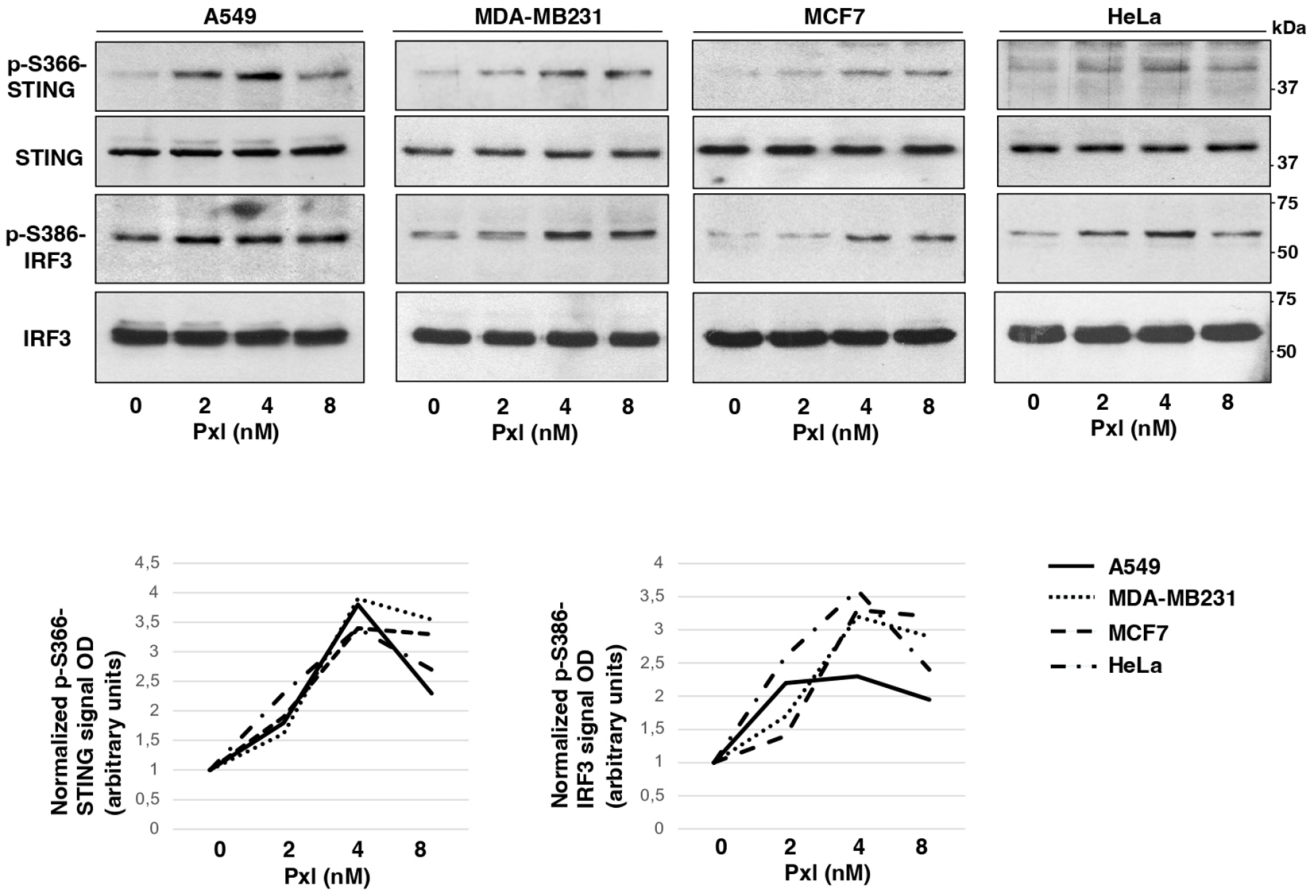
Pxl induces activating phosphorylations of STING and IRF3. A549, MDA-MB231, MCF7 and HeLa cells were treated for 48 hours with either vehicle (DMSO; 0 nM Pxl) or the indicated doses of Pxl (2, 4, 8 nM), lysed and proteins probed for the indicated antigens (the blots shown are representative of three independent experiments giving similar results). Lower graphs show a quantitation of optical density values of the phosphorylated STING and IRF3 signals normalized to the optical density values of the relative total protein signal.

Next, we analyzed whether Pxl treatment led to activation of a proinflammatory cascade in cancer cells. To this end we analyzed the phosphorylation status of STING at serine 366 (S366), a site known to be phosphorylated by TBK1 and required for further activation of IRF3, and the STING-TBK1-dependent, activating, phosphorylation of IRF3 at serine 386 (S386) upon treatment of A549, MDA-MB231, MCF7 and HeLa cells with increasing doses of Pxl in the low nanomolar range (2, 4 and 8 nM; **Figure 2**) (18, 19). Indeed, the Pxl treatment induced S366-STING and S386-IRF3 phosphorylations in all cell lines (**Figure 2**). The phosphorylated forms of STING and IRF3 tended to decline at 8 nM relatively to 4 nM Pxl treatment (**Figure 2**; see quantitative graphs of the phosphorylated forms). We do not have an exact explanation for this phenomenon, but we believe that it may possibly indicate that negative feedbacks, that attenuate excess proinflammatory signals, are initiated at the highest Pxl concentration (20).

### Pxl upregulates STAT1 phosphorylation as well as cGAS and PD-L1 protein levels in cancer cells

Following IRF3 activation, IFN type 1 genes are potently transcribed and IFN proteins produced and secreted (21). IFNs activate the JAK/STAT signaling cascade and phosphorylation of STAT1 at tyrosine 701 (Y701) is a hallmark of IFN pathway activation (22). Moreover, IFN and cGAS have been shown to be linked by positive feedback loops in which cGAS-dependent IFN induction further stimulates cGAS expression (23). In addition to the innate immunity-promoting action of cGAS, also recruitment of cytolytic CD8 T cells in the tumor microenvironment has been shown to be very dependent on cGAS activity (24). Considering that Pxl has also been shown to upregulate MHC class I, these effects of Pxl treatment may, indeed, strongly favor antitumor adaptive immunity (14).

Nevertheless, several lines of evidence also link the cGAS-STING pathway, STAT1 activation and IFN signaling to the upregulation of the expression of the immune checkpoint protein PD-L1 in cancer cells as well in cells of the immune system (25, 26). Thus, if the Pxl-activated cGAS/STING pathway would increase PD-L1 expression in cancer cells, this might promote, on the other hand, cancer cell escape from surveillance by the adaptive immune system (25, 26).

**Figure 3:**
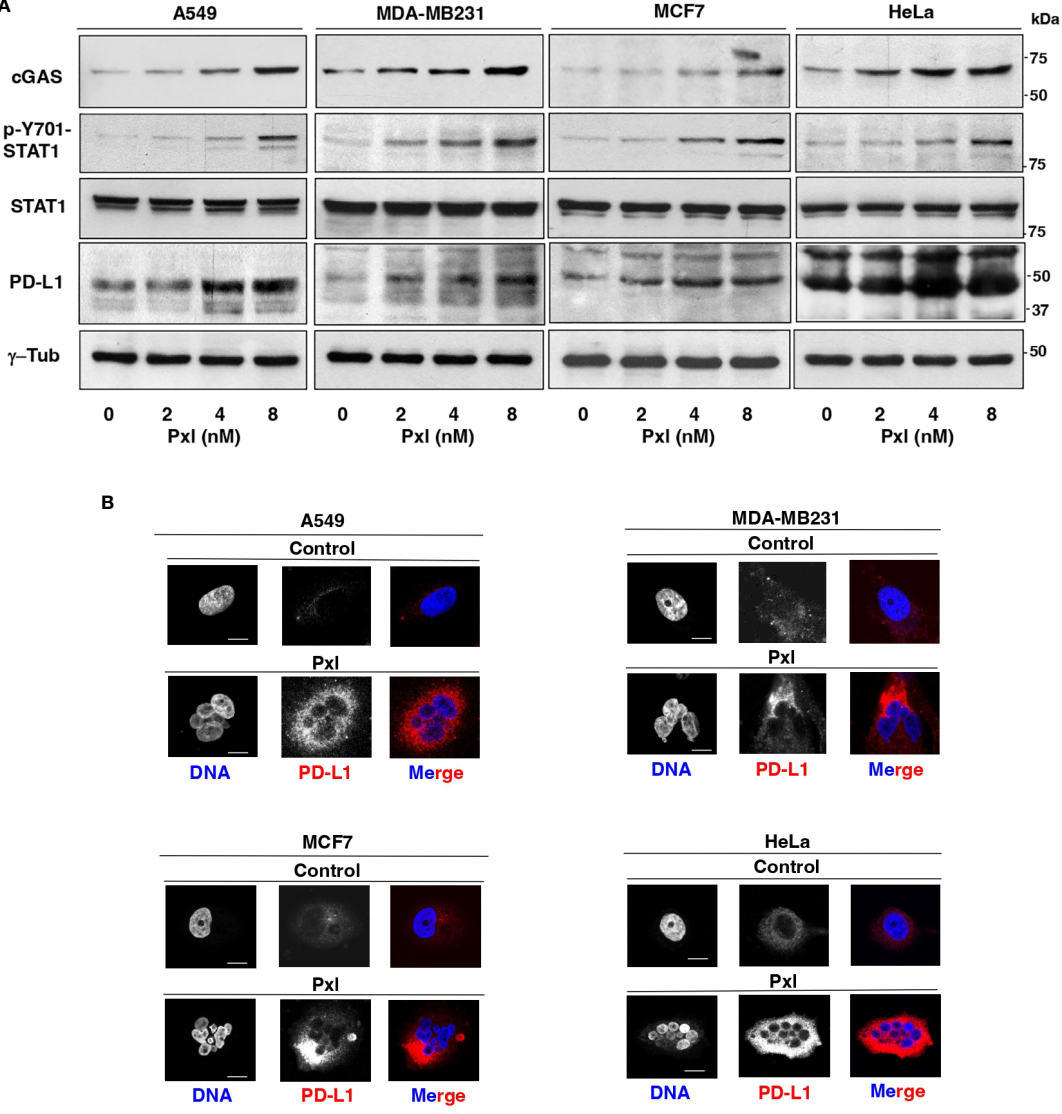
Pxl upregulates STAT1 phosphorylation and cGAS and PD-L1 protein levels in cancer cells. **(A)** A549, MDA-MB231, MCF7 and HeLa cells were treated for 48 hours with either vehicle (DMSO; 0 nM Pxl) or the indicated doses of Pxl (2, 4, 8 nM), lysed and proteins probed for the indicated antigens (the blots shown are representative of three independent experiments giving similar results). **(B)** A549, MDA-MB231, MCF7 and HeLa cells were treated for 48 hours with DMSO (Control) or 8 nM Pxl (Pxl). Cells were fixed and processed for indirect PD-L1 immunofluorescence and DNA staining. Scale bar: 5 μm.

We thus asked whether Pxl treatment led to Y701-STAT1 phosphorylation and upregulated cGAS and PD-L1 protein levels. To this end, A549, MDA-MB231, MCF7 and HeLa cells were treated for 48 hours with low doses of Pxl and the levels of phosphorylated Y701-STAT1 (p-Y701-STAT1) and of cGAS and PD-L1 protein analyzed (**Figure 3A**). Indeed, in all cell lines tested Pxl induced Y701-STAT1 phosphorylation, marker of IFN pathway activation, and significantly upregulated cGAS and PD-L1 expression (**Figure 3A**). In addition, Pxl treatment induced a substantial increase of PD-L1 at the cell surface in all cell types, as shown by PD-L1 immunofluorescence of non-permeabilized cells (**Figure 3B**).

### Pxl-induced upregulation of STAT1 phosphorylation and PD-L1 protein levels is cGAS-dependent

**Figure 4:**
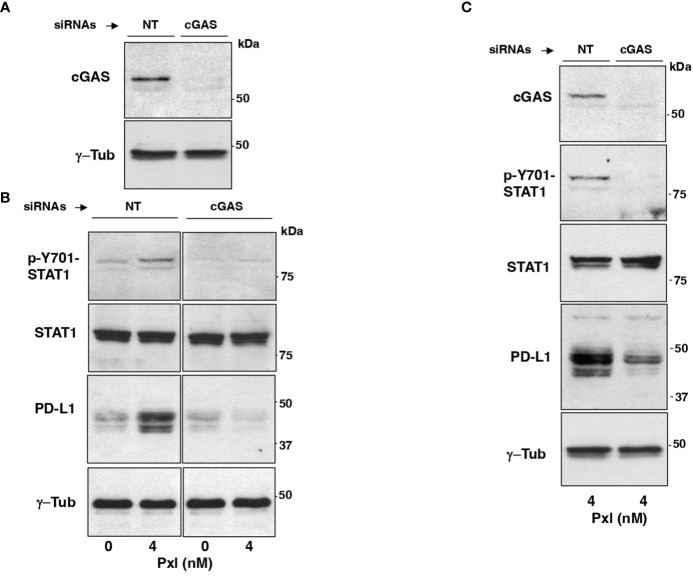
Pxl induction of STAT1 phosphorylation and PD-L1 upregulation require cGAS. **(A, B)** MDA-MB231 cells were treated with non-targeting (NT-siRNAs) or cGAS-targeting small interfering RNAs (cGAS-siRNAs) 24 hours before vehicle (DMSO; 0 nM Pxl) or Pxl (4 nM) addition, then, cells were taken after further 48 hours incubation. **(A)** The siRNAs treatment resulted in more than 90% downregulation of the cGAS protein expression in cGAS-siRNA-treated cells compared with NT-siRNAs-treated cells. **(B)** Lysates of NT-siRNA- or cGAS-siRNA-treated cells treated with vehicle (DMSO; 0 nM Pxl) or Pxl (4 nM) were probed for the indicated antigens. **(C)** A549 cells were treated with non-targeting (NT-siRNAs) or cGAS-targeting small interfering RNAs (cGAS-siRNAs) 24 hours before Pxl (4 nM) addition. Cells were further incubated for 48 hours, then, lysed and proteins probed for the indicated antigens. The blots shown are representative of three independent experiments giving similar results.

We asked whether Pxl-induced upregulation of STAT1 phosphorylation and of PD-L1 expression were mediated by the cGAS/STING pathway. To this end, MDA-MB231 cells were treated with non-targeting (NT-siRNAs) or cGAS-targeting small interfering RNAs (cGAS-siRNAs) 24 hours before Pxl (4 nM) or vehicle (Pxl 0 nM; as control) addition, then, cells were taken after further 48 hours incubation (**Figures 4A, B**). The siRNAs treatment resulted in more than 90% downregulation of the cGAS protein expression in cGAS-siRNA-treated cells compared with NT-siRNAs-treated cells (**Figure 4A**). Moreover, induction of Y701-STAT1 phosphorylation as well as the increase of PD-L1 protein expression induced by Pxl in control (NT-siRNA-treated) cells were completely blunted in the cGAS-downregulated (cGAS-siRNA-treated) cells, while the levels of total STAT1 protein were not affected by cGAS downregulation (**Figure 4B**). Similar results were obtained by downregulating cGAS expression in A459 cells under similar treatment conditions as described for MDA-MB231 cells (**Figure 4C**). These data indicate that IFN pathway activation and upregulation of PD-L1 protein in cancer cells treated with Pxl are indeed cGAS-dependent.

## Discussion

Recent evidence suggests that MTAs like Taxanes are efficient anticancer drugs because, in addition to directly kill cancer cells, they induce a proinflammatory response that may strongly contribute to their therapeutic effects (1, 14, 17, 27). By upsetting chromosome segregation and producing micronuclei or DNA bridges, Taxanes ultimately activate the cGAS/STING pathway that helps killing cancer cells through several inflammation-dependent mechanisms, including induction of higher sensitivity to apoptosis and increased recruitment of anticancer adaptive immunity (17, 25, 28). Indeed, the proinflammatory effects of Taxanes have stimulated the idea that their combination with ICIs could improve the therapeutic outcome. In fact, several clinical trials have shown some benefit from this therapeutic combination (27, 29, 30).

Our data from cancer cell cultures confirm, indeed, that Pxl promotes a cGAS-STING pathway-dependent proinflammatory cascade, but they also show that Pxl treatment induces a strong upregulation of the PD-L1 protein in cancer cells in a cGAS-STING pathway-dependent manner (see **Figures 3**, **4**). These observations are consistent with the view that negative feedbacks regulate the inflammatory response and with notion that the inflammatory response may have opposite effects on cancer development (20, 31). Indeed, the upregulation of PD-L1 expression by cancer cell is believed to substantially contribute to cancer cell resistance to immunosurveillance and to promote cancer cell growth and migration in an auto/paracrine manner as well (32). In addition, our data indicate that micronucleation, activation of a proinflammatory cascade and PD-L1 upregulation are induced by very low doses of Pxl (see **Figures 1**–**3**).

On the basis of these findings, we believe that combination therapy of Pxl, and possibly its derivatives, with ICIs should be tailored in way that takes into account that low doses of Pxl are indeed sufficient to induce a proinflammatory cascade, so that even poorly inflamed, “cold”, tumors can be turned into “hot” tumors increasing the recruitment of immune cells in the tumor microenvironment, but at the same time low Pxl doses are as well able to upregulate immune checkpoint proteins like PD-L1 that would, on the contrary, block adaptive immunosurveillance (33–35). In addition, although Taxanes have been shown to upregulate MHC molecules and reduce immunosuppressive T regulatory cells, thus favoring anticancer adaptive immunity, like other chemotherapeutic drugs, they are strongly myelosuppressive and may cause overall lymphopenia (36).

**Figure 5:**
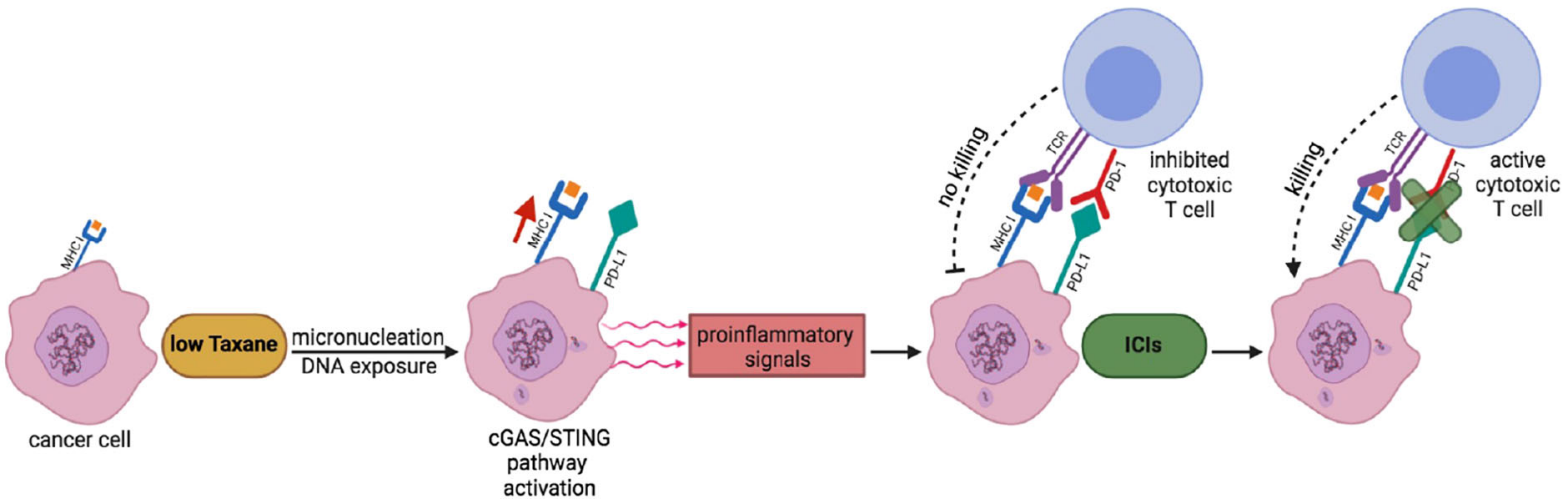
A scheme to potentiate Pxl + ICI combination therapy. Low Taxane dose induces micronucleation and cytosolic exposure of DNA. The following cGAS-STING pathway activation leads to release by cancer cells of proinflammatory signals that render “hot” the tumor microenvironment and help recruitment of cytotoxic T cells. At the same time, however, the cGAS-STING pathway leads to the upregulation of PD-L1 expression in cancer cells that inhibits T cell activation. The sequential treatment with ICIs unlocks the cancer cell killing potential of T cells.

Indeed, the Taxane/ICI combination therapy has been already tested in clinic and appears to result in beneficial effects (29, 30, 37, 38). Nevertheless, based on our findings, we would like to propose that the combination therapy may require further testing in terms of Taxane dosing and scheduling relatively to the ICI treatment in clinical trials to further improve clinical benefit (33–35, 39). In particular, we would like to propose that combination therapies with Pxl, and possibly its derivatives, and ICIs, especially anti PD-L1/PD targets, should be approached first by a low dosage, rather than near to maximum tolerated, Pxl regimen in order to render “hot” the tumor microenvironment and to “prime” cancer cells for a sequential, rather than concurrent, treatment with ICIs (see **Figure 5**) (14, 15, 27, 35, 39). In addition, our data further support the evaluation of cGAS levels as a biomarker, that could be more technically reliable than evaluation of PD-L1 itself, to predict beneficial effects of an ICI adjuvant treatment when Pxl and derivatives are used in neodjuvant setting (40).

## Data availability statement

The original contributions presented in the study are included in the article/**Supplementary Material**. Further inquiries can be directed to the corresponding author.

## Author contributions

Conceptualization, AS and DG. Methodology, experimental work, AS and CP. Writing, original draft preparation, DG. Writing, review and editing, AS, CP and DG. Supervision, DG. All authors contributed to the article and approved the submitted version.
