# Supplementary material for: cGAS-dependent proinflammatory and immune homeostatic effects of the microtubule-targeting agent paclitaxel
Source: Front Immunol. 2023 Mar 7;14:1127623. doi: 10.3389/fimmu.2023.1127623 (PMC10028148; doi:10.3389/fimmu.2023.1127623)
Supplement: Supplementary file 1 [file DataSheet_1.pdf]

cGAS-dependent proinflammatory and immune homeostatic effects of the Microtubule-Targeting Agent Paclitaxel

Angela Flavia Serpico, Caterina Pisauro, Domenico Grieco *

*** Correspondence:** Domenico Grieco; domenico.grieco@unina.it

Supplementary Figures

A

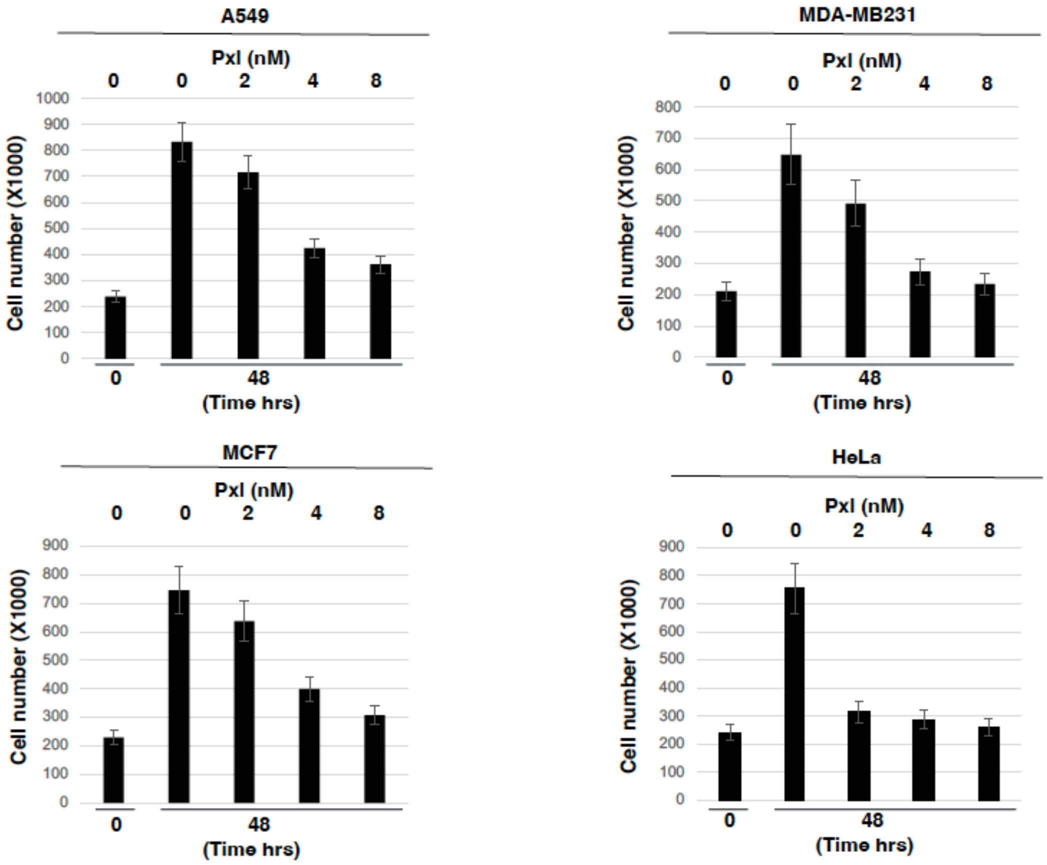

B

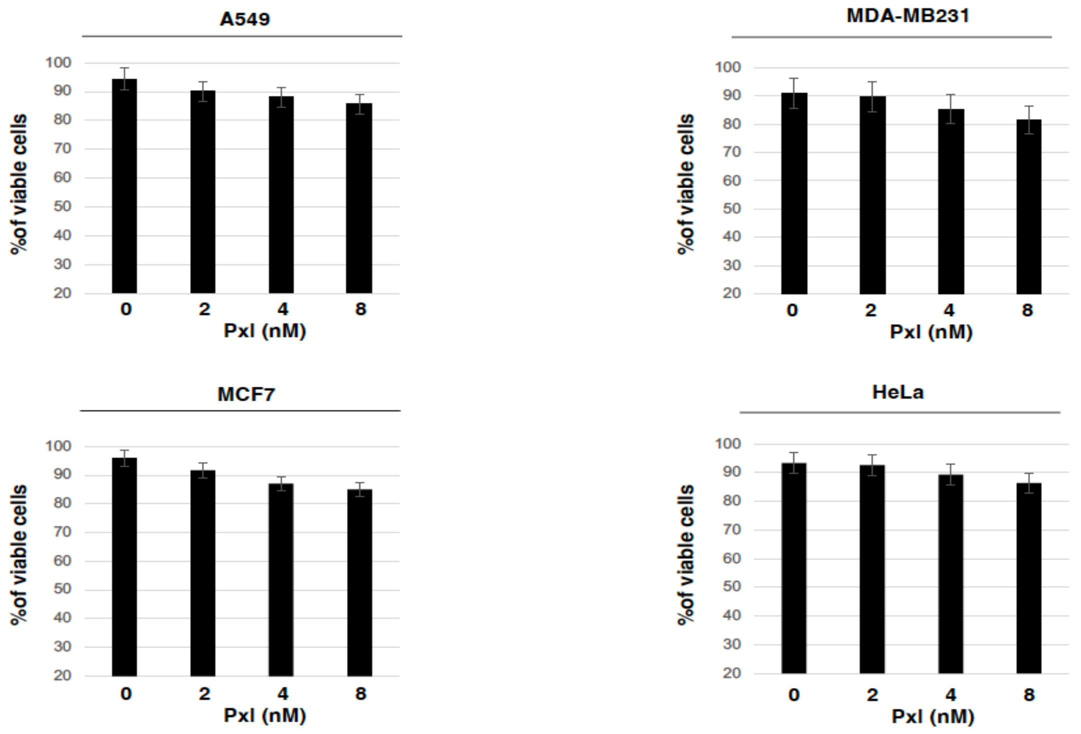

Supplementary Figure S1. Cytostatic effects of Pxl. (**A**) Cells were seeded at a cell density of 7000/cm^2. The next day, one cell sample was counted (Time 0) and the other cell samples were treated with either vehicle (DMSO) as control (0 nM Pxl) or with 2, 4 and 8 nM Pxl. Cells were then counted after further 48 hours of incubation. (**B**) The graph reports the percent of viable cells, scored by trypan blue exclusion, after 48 hours incubation with vehicle (DMSO; 0 nM Pxl) or 2, 4 and 8 nM Pxl. Error bars refer to variability within triplicate samples.

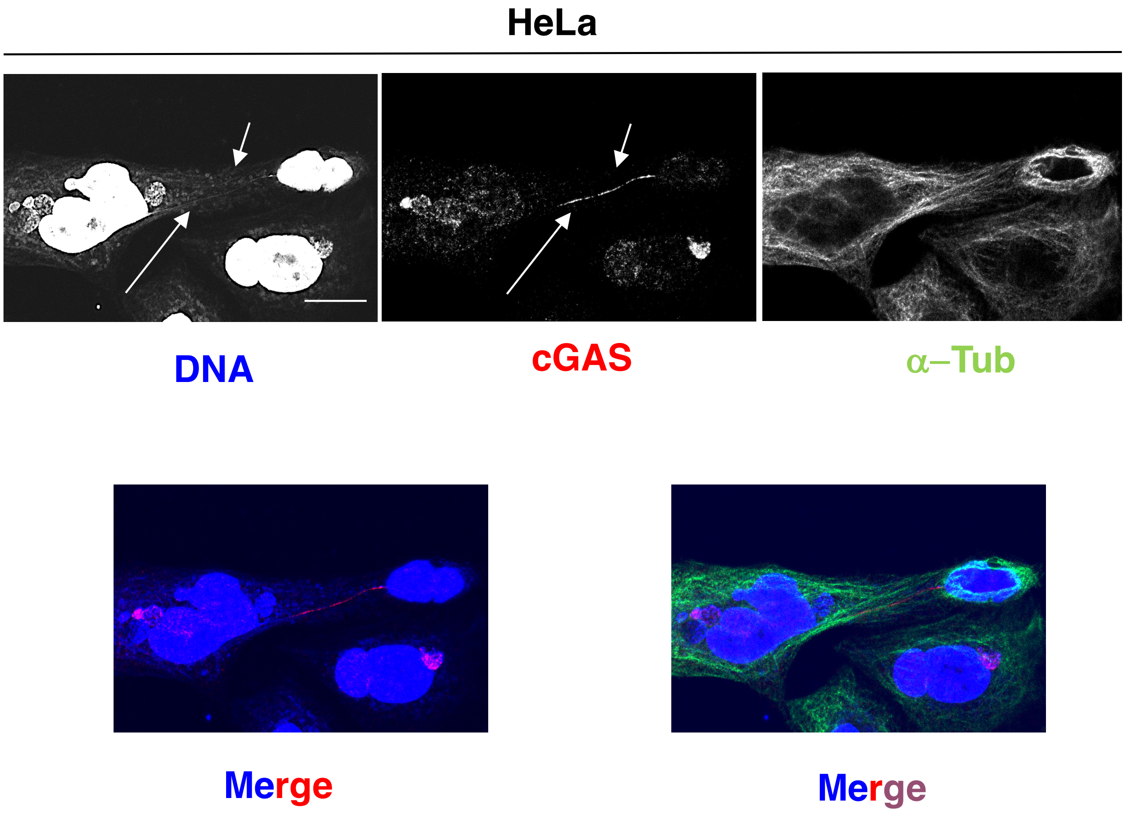

Supplementary Figure S2. Pxl induces formation of cGAS-positive chromatin bridges. HeLa cells were treated for 48 hours with Pxl (4 nM) fixed and processed for indirect immunofluorescence (IF) staining for the indicated antigens (cGAS in red; α-tubulin (α-Tub) in green; DNA in blue. Arrowheads indicate cGAS-positive chromatin bridges.
